# Human Stiff-Person Syndrome IgG Induces Anxious Behavior in Rats

**DOI:** 10.1371/journal.pone.0016775

**Published:** 2011-02-08

**Authors:** Christian Geis, Andreas Weishaupt, Benedikt Grünewald, Thomas Wultsch, Andreas Reif, Manfred Gerlach, Ron Dirkx, Michele Solimena, Daniela Perani, Manfred Heckmann, Klaus V. Toyka, Franco Folli, Claudia Sommer

**Affiliations:** 1 Department of Neurology, University of Würzburg, Würzburg, Germany; 2 Department of Psychiatry, University of Würzburg, Würzburg, Germany; 3 Department of Child and Adolescent Psychiatry, Psychosomatics and Psychotherapy, University of Würzburg, Würzburg, Germany; 4 Institute of Physiology, University of Würzburg, Würzburg, Germany; 5 Rudolf Virchow Center, DFG Research Center for Experimental Biomedicine, University of Würzburg, Würzburg, Germany; 6 Molecular Diabetology, Carl Gustav Carus Medical School, Dresden University of Technology, Dresden, Germany; 7 Division of Neuroscience, Vita-Salute San Raffaele University, Milan, Italy; 8 Division of Neuroscience, Department of Nuclear Medicine, Scientific Institute San Raffaele, Milan, Italy; 9 Diabetes Division, Department of Medicine, University of Texas Health Science Center, San Antonio, Texas, United States of America; University of Muenster, Germany

## Abstract

**Background:**

Anxiety is a heterogeneous behavioral domain playing a role in a variety of neuropsychiatric diseases. While anxiety is the cardinal symptom in disorders such as panic disorder, co-morbid anxious behavior can occur in a variety of diseases. Stiff person syndrome (SPS) is a CNS disorder characterized by increased muscle tone and prominent agoraphobia and anxiety. Most patients have high-titer antibodies against glutamate decarboxylase (GAD) 65. The pathogenic role of these autoantibodies is unclear.

**Methodology/Principal Findings:**

We re-investigated a 53 year old woman with SPS and profound anxiety for GABA-A receptor binding in the amygdala with (11)C-flumazenil PET scan and studied the potential pathogenic role of purified IgG from her plasma filtrates containing high-titer antibodies against GAD 65. We passively transferred the IgG fraction intrathecally into rats and analyzed the effects using behavioral and *in vivo* electrophysiological methods. In cell culture, we measured the effect of patient IgG on GABA release from hippocampal neurons. Repetitive intrathecal application of purified patient IgG in rats resulted in an anxious phenotype resembling the core symptoms of the patient. Patient IgG selectively bound to rat amygdala, hippocampus, and frontal cortical areas. In cultured rat hippocampal neurons, patient IgG inhibited GABA release. In line with these experimental results, the GABA-A receptor binding potential was reduced in the patient's amygdala/hippocampus complex. No motor abnormalities were found in recipient rats.

**Conclusion/Significance:**

The observations in rats after passive transfer lead us to propose that anxiety-like behavior can be induced in rats by passive transfer of IgG from a SPS patient positive for anti-GAD 65 antibodies. Anxiety, in this case, thus may be an antibody-mediated phenomenon with consecutive disturbance of GABAergic signaling in the amygdala region.

## Introduction

Anxiety and fear are the leading symptoms in anxiety disorders such as panic disorder and phobias, which are thought to feature complex neurobiological underpinnings with both genetic as well as environmental factors. Cortico-limbic pathways and GABAergic signaling are thought to be key components of anxiety disorders, yet the precise molecular mechanisms are still unknown. In addition to anxiety disorders in a narrower sense, anxious behavior can frequently be found in a wide range of neuropsychiatric diseases. Although the pathomechanisms are even less clear in these disorders, it can be supposed that disease mechanisms overlap and a common final pathway may exist. Stiff person syndrome (SPS), a rare and multi-facetted disorder of the central nervous system, is one of the neuropsychiatric disorders where anxious symptoms are found most frequently [1], [2]. The most prominent and eponymous clinical feature of this complex syndrome is motor hyperexcitability leading to increased muscle stiffness and intermittent muscle spasms [3], [4], which has been attributed to decreased GABAergic inhibition at the spinal cord and brainstem level [5]. However, the anxious phenotype of the patients which often resembles agoraphobia and not rarely entails substance abuse, often leads to a misdiagnosis of a primary psychiatric disorder and must clearly be attributed to supraspinal pathology. This may be one reason why SPS remains still an underdiagnosed condition [6].

It is a matter of debate whether anxiety and agoraphobia may be secondary to the motor instability caused by the enhanced startle response associated with uncontrolled drop attacks, or if these are additional and autonomous symptoms reflecting central GABAergic dysfunction [7], [8], [9]. There is ample evidence that anxiety disorders are related to disturbances in the GABAergic system in distinct brain regions, such as the amygdala, hippocampus, or frontal cortex [10], [11], [12], [13]. Moreover, GABA-A receptor binding is reduced in patients with panic disorder and other anxiety-related disorders [14], and mice deficient of GAD 65 have deficits in consolidation and generalization of fear memory [15], [16]. Autoantibodies against glutamate decarboxylase 65 (GAD 65) in turn are found in up to 80% of patients with the non-paraneoplastic form of SPS [17], [18], [19]. Here we re-evaluate a female patient reported as a clinical case of SPS earlier and describe the anxiety-like behavior upon intrathecal passive transfer of IgG in the rat reproducing one of the core signs of human SPS.

## Results

### Reduced (11)C-FMZ PET binding potential of the amygdala and hippocampal complex

**Figure 1:**
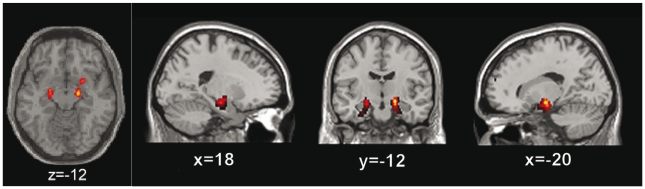
Bilateral reductions of (11)C-FMZ binding potential in amygdala regions in the SPS patient with GAD 65 autoantibodies. This figure shows the results of the statistical parametric mapping (SPM) with voxel-based analysis and thus the comparison of GABA-A receptor binding potential in the patient when compared to normal controls. Note that the (11)C-FMZ binding potential is significantly reduced bilaterally in the patient's amygdala region.

The PET scan of a 53-year-old woman with SPS reported earlier as a clinical case [20] was re-evaluated. In brief, the patient showed high rates on the Hamilton Anxiety Rating Scale and Zung Self-Rating Anxiety Scale scale, and in the anxiety dimension of the Symptom Checklist-90. There were no other neuropsychological deficits nor significant levels of depression (Table 1), and the patient was not on pharmacological treatment at the time of PET studies. The PET scan analysis showing reduced ^11^C-FMZ binding potential in motor-premotor cortex was now extended and we show that the binding potential was also reduced in the limbic region, namely the amygdala and hippocampal complex (Figure 1; right: 1.80 vs. 2.18±0.39, left: 1.58 vs. 1.96±0.41, SPS patient vs. control patients, means ± SD).

**Table 1:** Neuropsychological evaluation of the SPS patient.

test	test result SPS patient	interpretation
HARS	16	(0–56)significant anxiety (>14)
HAM-D	0	(0–66)mild depression (>18)
MADRS	1	(0–60)mild depression (>9–18)
SAS	51	(0–80)moderate anxiety level (45–59)
SCL-90	anxiety: 28	(item 5: 0–40)significant anxiety: (>20)
MMSE	30	(0–30)no cognitive impairment

Neuropsychological evaluation of the SPS patient [20] revealed abnormal ratings on the Hamilton Anxiety Rating Scale (HARS), the anxiety subscale of the Symptom Checklist-90 (SCL-90), and the Zung Self-Rating Anxiety Scale (SAS) indicating clinical significant anxiety. There was no indication of depression or cognitive impairment as tested with the Hamilton Depression Scale (HAM-D), Montgomery-Asberg Depression Scale (MADRS), and Mini-Mental State Examination (MMSE). The range of the respective test scores are given in brackets.

### Intrathecal passive transfer of SPS patient IgG induces anxiety-like behavior

**Figure 2:**
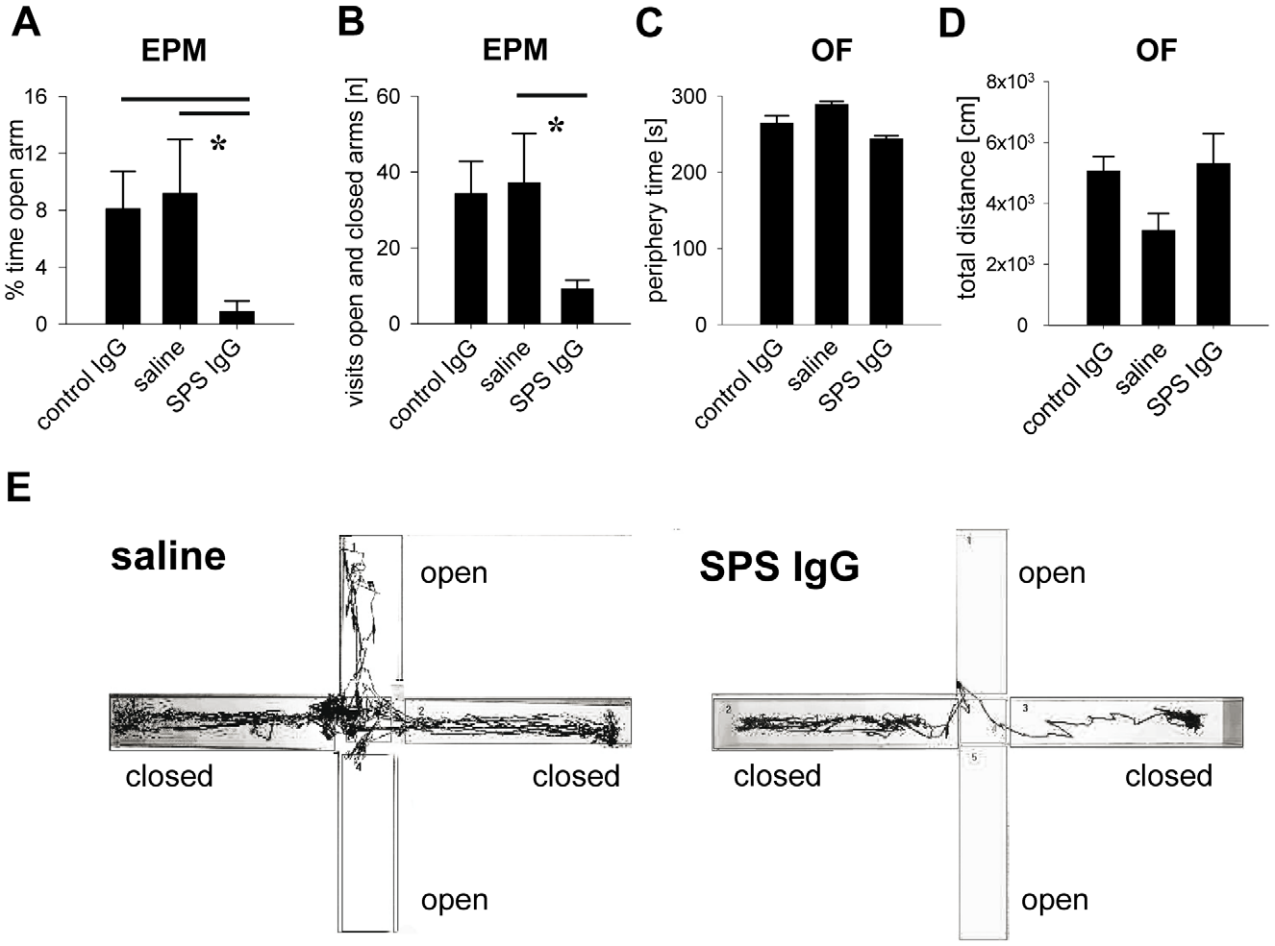
SPS patient IgG induces anxiety-like behavior. (A and B) Animals after intrathecal passive transfer of IgG were tested on an elevated plus maze (EPM) to analyze anxiety-like behavior. Rats treated with SPS patient IgG (SPS IgG; n = 6) moved normally but spent significantly less time in the open arms and explored significantly less arms of the EPM in testing period compared to controls (control IgG: n = 9; saline n = 7), indicating increased anxiety-like behavior (* p<0.05; Mann-Whitney Test). (C and D) Locomotor activity, assessed as time spent in the periphery of the open field (OF) and total distance moved during the observation period, was not different between the experimental groups and did not contribute to the behavioral observations in the EPM. (E) Representative tracks of a rat treated with saline (left panel) and SPS IgG (right panel) in the EPM. Whereas the control animal explored 3 arms of the EPM including one open arm with bright illumination, the rat treated with SPS IgG stayed in the closed, darker arms and avoided entries into the open arms.

Because the patient was clinically affected by profound anxiety, agoraphobia, and panic attacks, we focused on anxiety-like behavior in experimental rats receiving repetitive i.th. injections of purified patient IgG. Indeed, in the elevated plus maze (EPM), rats treated with GAD 65 antibody positive IgG showed pronounced anxiety-like behavior. The animals preferred to stay in the closed arms of the maze, spent less then 1% of the testing time in the open arms and made significantly less entries in either arm, when compared to controls (Figures 2A, B, E). Differences in activity did not contribute to these behavioral observations, because the activity levels in the open field (Figure 2C and D) were not different between the experimental groups. None of these behavioral changes were seen with control patient IgG fractions.

In contrast to observations made in an acute *ex-vivo* preparation by others [21], i.th. spinal application of human SPS IgG from our patient into the living rat did not induce muscle spasms or stiffness. Behavioral testing of the motor system including RotaRod analysis, forelimb grip strength or gait analysis did not reveal any motor deficits when compared to controls (data not shown). We then searched for subclinical motor abnormalities, but the *in-vivo* electrophysiology as established in another treatment protocol [22], [23] did not reveal abnormal spinal transmission in all three experimental groups. Post-activation depression of the H-reflex was not reduced and the H-reflex was almost completely inhibited at a10 Hz stimulation in all tested animals as in controls (Figure S1A). Moreover, the time-dependent D1/D2 inhibition of the H-reflex resembling GABAergic suppression of heteronymous muscle activation due to primary afferent depolarization (PAD) was intact and not significantly different in all experimental groups with the maximal depression in an interstimulus-interval between 25 and 100 ms (Figure S1B). Measuring PAD directly by recording the dorsal root potentials of the treated animals *in-vivo* revealed no differences between the experimental groups, both at single stimulation and at repetitive stimulation at higher frequencies (Figure S1C).

### Patient IgG binds to rat and human GABAergic structures in the CNS

**Figure 3:**
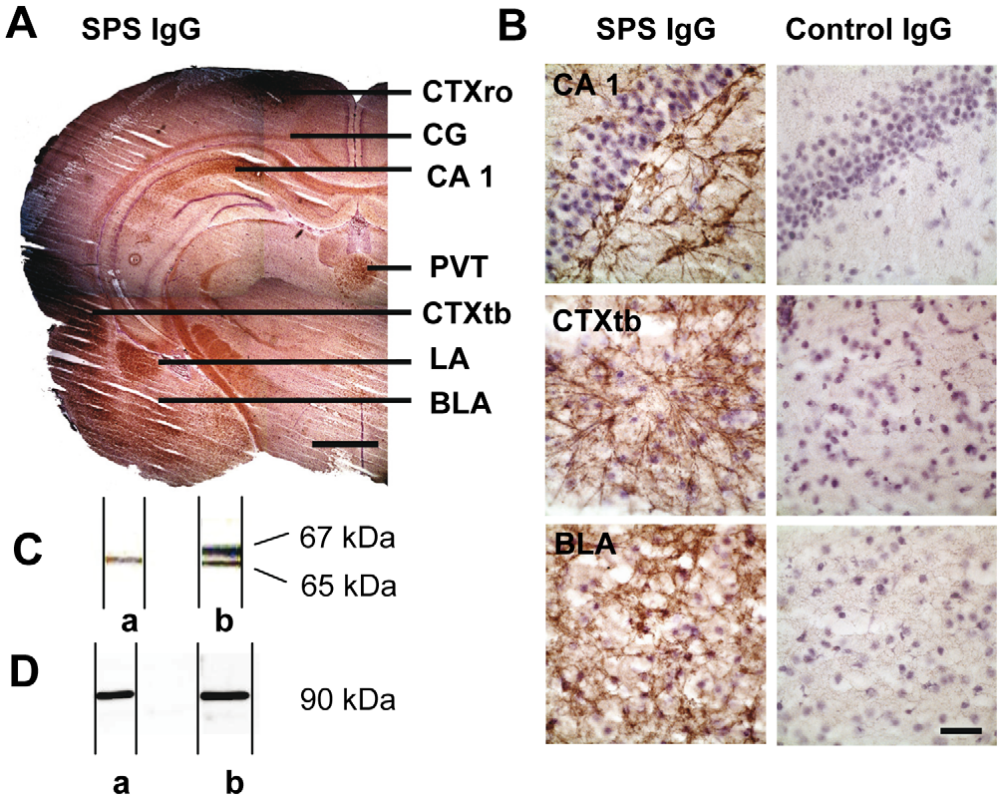
Purified SPS-IgG binds selectively to defined areas in rat brain. (A) Incubation of naïve rat brain sections with purified patient SPS IgG resulted in distinct labeling of different brain areas, such as the rostral and temporobasal part of the cortex (CTXro, CTXtb), hippocampus (HC, CA1), nucleus paraventricularis thalami (PVT), lateral and basolateral amygdala (LA, BLA), whereas in other areas, e.g. most of the thalamus, parts of the cortex and midbrain regions, staining was absent or less pronounced (scale bar: 2.0 mm). (B) At higher magnification, the staining pattern differed within the labeled brain regions. In the HC strongest immunoreactivity was detected in the CA1 region just below the pyramidal cell layer with clear labeling of single neurons resembling GABAergic basket neurons. In the immunoreactive areas of the cortex (temporobasal part: CTXtb), labeling of single neurons with extensive staining of the dendrites was observed. The strongest immunoreactivity was found in the region of the amygdala nuclei, most pronounced in the lateral and basolateral parts (basolateral part: BLA) with dense reticular staining and strongly immunoreactive small cell bodies showing a dense network within the amygdala nuclei. No specific staining was detectable after incubation with control IgG (scale bar: 25 µm). (C) Western blotting of patients purified IgG on rat CNS tissue revealed a single band at 65 kDa (lane a) while the characteristic double band at 65 and 67 kDa was seen when a commercial polyclonal GAD 65/67 antibody was used (lane b). (D) Western blotting of patient purified IgG (lane a) and a rabbit polyclonal GAD 65 antibody (lane b) on a rat GAD 65 fusion protein displayed specific binding with a single band at the expected molecular weight of 90 kDa.

**Figure 4:**
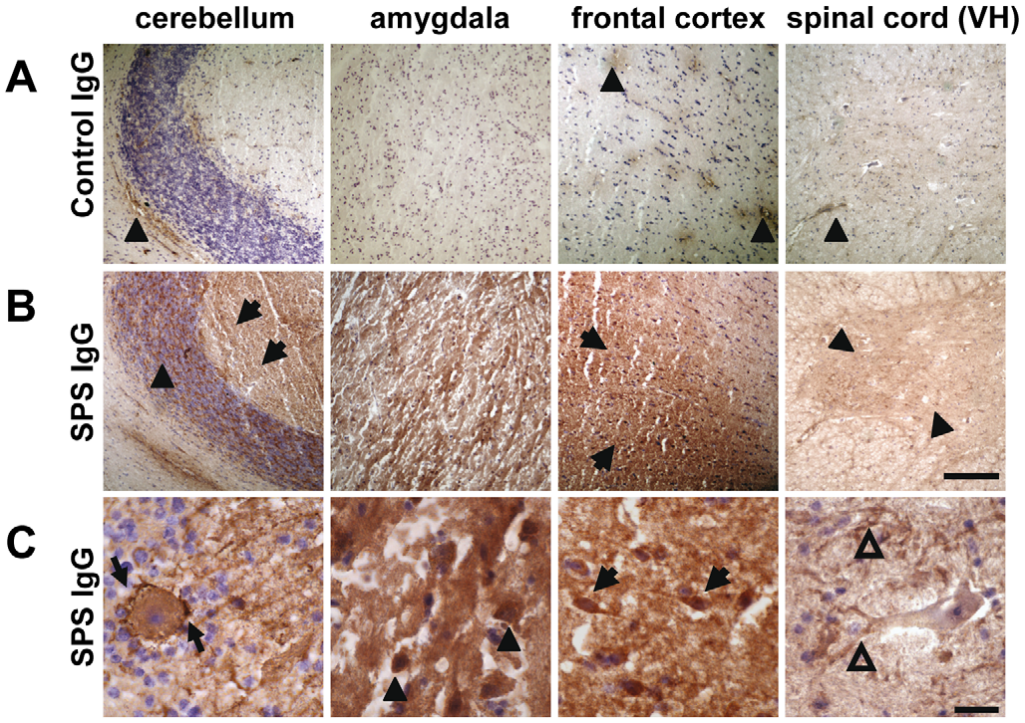
Binding properties of purified SPS - IgG on human CNS tissue. Tissue of normal human cerebellum, amygdala, frontal cortex, and spinal cord was immunoreacted with purified control IgG or IgG preparations containing high titer of GAD 65 antibodies (scale bar 200 µm). Incubation with control IgG (A) resulted in unspecific perivascular staining (arrowheads), whereas SPS IgG (B) gave intense labeling of the molecular (arrows) and granular layer (arrowheads) in the cerebellum, of the amygdala core region, and the grey matter of the frontal cortex (arrows). SPS IgG immunoreactivity in the ventral horn (VH) of the spinal cord (arrowheads) was less pronounced as compared to the highly immunoreactive brain regions, particularly the amygdala and frontal cortex. (C) Higher magnification revealed (1) strongly positive staining of GABAergic basket cell fibers around Purkinje cells in the cerebellum (thin arrows), (2) a very intense staining of the densely packed reticular network in the amygdala and some small sized interneurons (arrowheads), (3) positive immunoreactivity of single interneurons (thick arrows) in the deeper layers of the frontal cortex, and (4) less strong staining of perineuronal dendrites and proximal dendrites of a motor neuron (open arrowheads) in the ventral horn of the spinal cord (scale bar: 25 µm).

Western blotting with homogenized rat CNS tissue (Figure 3C) and with a fusion protein of rat recombinant GAD 65 protein (Figure 3D) gave distinct bands with the IgG preparation indicating that the patient's IgG antibodies detected rat GAD 65. Rat CNS sections displayed a pronounced immunoreactivity of distinct areas when incubated with purified SPS patient IgG, in particular in structures associated with emotional control, such as the amygdala-hippocampal complex, frontal cortex, and limbic structures (Figure 3). Moreover, when we used human autopsy CNS material, similar staining patterns were observed (Figure 4). In contrast, control IgG gave only faint background staining.

**Figure 5:**
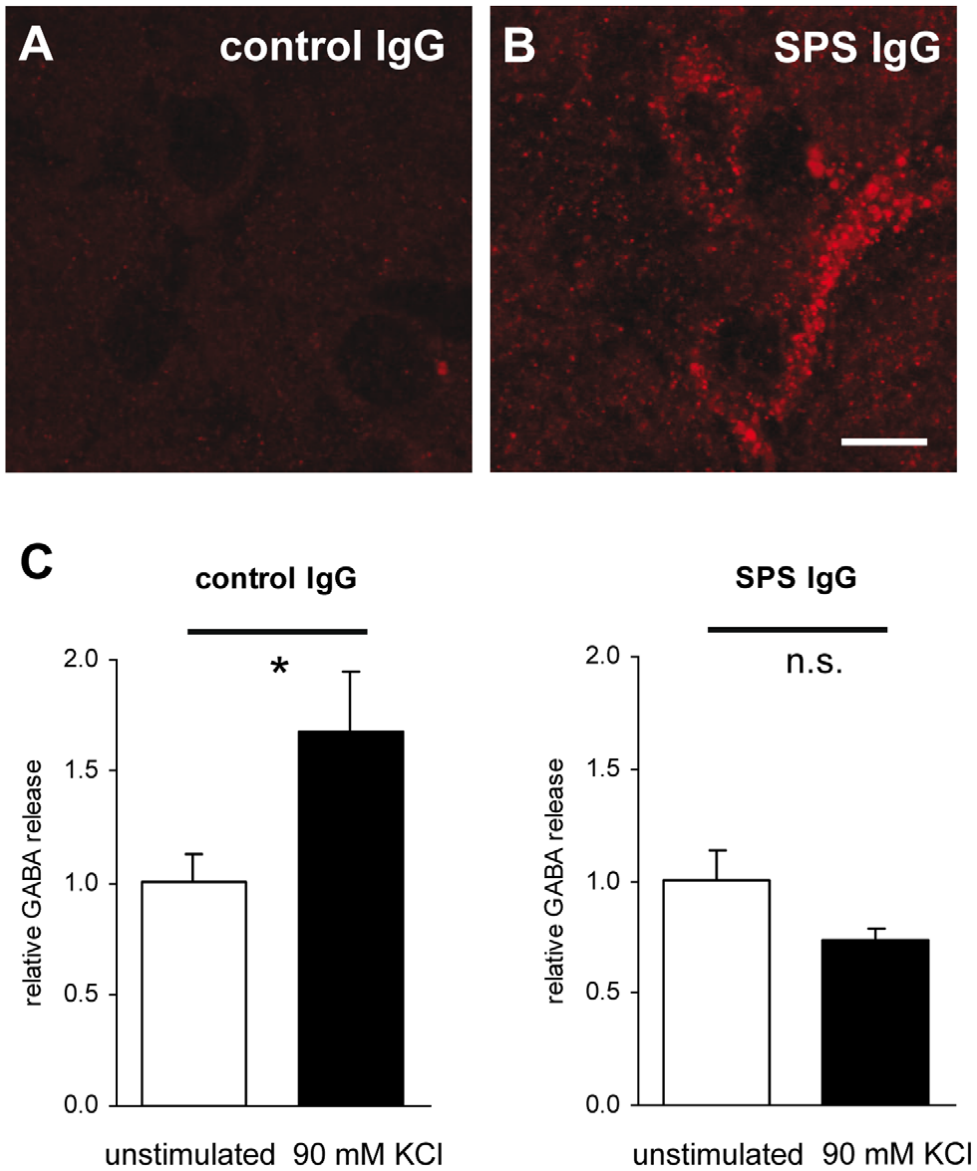
SPS IgG accumulates in interneurons of rat amygdala complex and reduces GABA release from hippocampal neurons. In rats treated intrathecally with SPS IgG (A) but not in those treated with control IgG (B), immunoreaction against human IgG showed positive staining of neurons in the amygdala complex (scale bar: 10 µm). (C) Dissociated hippocampal neurons from E18 mouse embryos were incubated with SPS or control IgG. The increase of GABA release into the supernatants induced by stimulation with 90 mmol KCl was absent in SPS IgG treated neuronal cell cultures as demonstrated by HPLC analysis (* p<0.05, Student's t-test).

When brain sections of the intrathecally treated rats were stained for human IgG, strongly immunoreactive neurons were found in the basolateral amygdala, suggesting neuronal uptake of patient IgG in the amygdala complex. These findings also demonstrate that the i.th. injected IgG had indeed access to the CNS hemispheres. No immunoreactivity was found in the animals treated with control IgG (Figure 5A and B). We found no deposition of activated complement in brain sections of the experimental animals and we could not detect any obvious signs of neuronal cell death (data not shown), making an antibody dependent activation of the complement system with or without subsequent cell destruction an unlikely mechanism.

### Patient IgG reduces stimulated GABA release from cultured hippocampal neurons

To assess a direct effect of patient IgG on GABAergic transmission, we measured GABA release in the culture supernatant of cultured hippocampal neurons with high performance liquid chromatography (HPLC). Incubation with patient SPS IgG resulted in a significant decrease of GABA release after stimulation with 90 mmol KCl compared to incubation with control IgG (Figure 5C).

## Discussion

The principal finding of our study is that one of the core symptoms – namely, anxiety – of the afflicted SPS patient could be reproduced in the recipient rat by passively transferring her GAD 65 antibody-containing IgG into the subarachnoid space. This points to a probable pathogenic action of IgG autoantibodies. Furthermore, application of IgG at the caudal spinal cord lead to distant IgG binding at the amygdala that is likely linked to the observed behavioral alterations.

How do our findings relate to the neuropsychiatric observations in the patient? The extension of the PET study of the SPS patient, showing a reduced ^11^C-FMZ binding potential in limbic structures, supports the concept that the GABAergic system in the amygdala region is affected by the disease, and this is consistent with a significant reduction in GABA-A receptor binding observed in patients with panic disorder and other anxiety-related disorders [14]. The PET findings suggest a dysfunction of the amygdala which may be interpreted as a disturbance of GABA signaling in patients with SPS, rather than a secondary effect due to repeated falls [13]. These findings are in accordance with previous reports of decreased GABA levels in selected brain regions measured with MRI spectroscopy [24] showing that GABAergic transmission is not uniformly affected in the brain of patients with SPS.

Based on our passive transfer experiments we like to propose that the neuropsychiatric changes may be a direct consequence of circulating IgG autoantibodies to GAD 65 although it cannot be excluded that autoantibodies with other as yet unknown specificities are instrumental in causing the behavioral abnormalities and IgG binding in recipient rats (see below).

The GABAergic involvement of the limbic cortex shown here might be due to the high demand of modulatory control in these regions, based on their finely tuned GABAergic transmission [25]. Our findings are also supported by and in good agreement with reports about increased fear-related behavior and altered responses to anxiolytic drugs in GAD 65 knockout mice, resulting from a desynchronization in the amygdala-hippocampal network [15], [26]. Furthermore, mice heterozygous for the γ2 subunit of the GABA-A receptor, with reduced synaptic clustering of the GABA-A receptor, showed an exaggerated anxiety behavior [10], [11].

In contrast to a recent report on the effects of GAD 65 antibody containing IgG in an acute ex-vivo preparation [21] and to our own studies with SPS derived antibodies against amphiphysin [22], we could neither observe acute motor hyperexcitability or muscle stiffness, nor a chronic motor disorder after repetitive applications of the IgG. The absence of a motor phenotype was corroborated by the results of *in-vivo* electrophysiological recordings including sensitive tests such as H-reflex activity and measure of presynaptic afferent depolarization [27] directly by recording the dorsal rot potential amplitudes. Thus, from our present data we have no direct evidence indicating that our SPS patient's IgG also lead to a diminished GABAergic inhibition in the rat spinal cord. The discrepancy between our findings and those in the ex-vivo preparations [21] is most likely resulting from the different experimental paradigms. Our results are in contrast to our earlier observations with antibodies against amphyphysin. We assume that the mechanism of disturbed vesicular endocytosis as observed by our group [22] may be a more ubiquitous one when compared to the effects of GAD 65 antibodies. In contrast, GAD65 dysfunction may be the consequence of a predominant vulnerability of neuronal pathways in the amygdala-hippocampal complex, according to the findings in the animal models with genetic modification of GAD65 expression (15;26; see above).

One question that still needs to be solved is whether the anti-GAD 65 antibodies in the patient's purified IgG fraction are directly responsible for the observed effects, or whether antibodies directed against yet unidentified surface antigens of GABAergic neurons might play a role. Using western blotting, we could not detect any reactivity towards antigens other than GAD65. However, as in the example of GABA-A-receptor-associated protein (GABARAP), identified as an additional antigen in patients with anti-GAD-positive SPS [28], the possibility of additional autoantibody specificities cannot be excluded. Using patient IgG depleted of anti-GAD65 IgG in animal models and in-vitro assays would be the most direct way to address this, but attempts to produce sufficient quantities of soluble and stable recombinant human GAD65 were not successful as also found by other groups [21], [29]. Since GAD65 is a cytosolic enzyme, which is not exposed at the cell surface, anti-GAD65 antibodies would have to enter the cells and obtain access to their antigen to exert a pathogenic role. Recently we have shown that antibodies to amphiphysin, another intracellular antigen in SPS patients, can indeed be taken up by neurons and bind to their intracellular protein antigen [22]. For GAD 65 antibodies this remains to be shown.

Recently a new family of paraneoplastic and idiopathic CNS disorders with neuropsychiatric symptoms, namely limbic encephalitis with psychiatric features, SPS, hyperexplexia, epilepsy and various other optional features has been identified to be linked to autoantibodies directed at CNS autoantigens including NMDA-receptors, AMPA-receptors, Caspr2 and LGI-1; some of these were formerly classified as voltage-gated potassium channelopathies [30], [31], [32], [33]. Many of these patients respond to immunosuppressive treatment and plasma exchange as described in GAD 65 and amphiphysin antibody-associated SPS opening up a wide field of antibody-mediated neuropsychiatric disorders.

Further studies will be necessary to confirm our findings in a larger number of patients. Another aim for follow-up studies is to identify the exact target of the SPS antibodies responsible for these observations. Taken further, our findings may imply that autoantibodies should be searched for in other anxiety related disorders, and, if detected, this may open new diagnostic and therapeutic options for patients with anxiety syndromes.

## Materials and Methods

### Patient

The experimental study was performed with purified IgG containing antibodies to GAD 65 obtained from a 53-year-old woman with SPS, who first presented with signs of muscle rigidity and spasms at age 52. Besides the typical motor symptoms, she suffered from severe anxiety, panic attacks, and agoraphobia [20]. In brief, the patient was examined clinically and by a detailed neuropsychological exploration including the Hamilton Anxiety Rating Scale (HARS), Hamilton Depression Scale (HAM-D), Montgomery-Asberg Depression Scale (MADRS), Zung Self-Rating Anxiety Scale (SAS), Symptom Checklist-90 (SCL-90), and Mini-Mental State Examination (MMSE) scales. For the purpose of this experimental study, her (11)C-flumazenil (FMZ) PET scan reported previously [20] was re-evaluated for central GABA-A receptor binding potential. Reconstructed images were re-analyzed using voxel-wise comparison of the ^11^C-FMZ binding potential in spatially normalized images of the patient with healthy controls (11 subjects, mean age: 31.4±11.9 years; range 24–60 years) [34]. The patient gave written consent for the publication of clinical details and results of examinations.

### Purification of the antibody-containing IgG fraction

Patient IgG and IgG from a control patient with chronic inflammatory polyneuropathy was purified from plasma filtrate obtained at therapeutic plasma exchange as a part of standard patient care by separation on exchange chromatography as described [23]. Both subjects gave written consent for further utilization of their plasma filtrate for experimental studies. Titers of anti-GAD 65 antibodies were measured by a commercial enzyme immunodot assay with rabbit antisera raised against recombinant GAD 65 as positive control. The titre of patient's anti-GAD 65 antibodies in the plasma filtrate was 130.210 mU/ml (normal values <70 mU/ml). The IgG fractions were dialyzed, freeze dried and stored at −20°C. Lyophilized IgG was dissolved in normal saline just before use and checked by Western blot.

The GAD 65 gene was sub-cloned into a pGEX-6P expression vector system (GE Healthcare, Munich, Germany) to generate a glutathione S-transferase (GST) fusion protein, which was transformed into DH5alpha cells. For Western blot analysis, 10–50 µg GAD 65 fusion protein was resuspended in 30 µl Laemmli sample buffer, fractionated by 10% SDS-PAGE and electroblotted onto a nitrocellulose membrane. Nitrocellulose was incubated with a polyclonal rabbit anti-GAD 65 antibody (1∶500, Acris, Hiddenhausen, Germany), a polyclonal anti-GAD 65/67 antibody (1∶500, Chemicon, Millipore, MA, USA) and patient IgG (100mg/ml, 1∶500). The proteins were detected by chemiluminescence with Supersignal West Pico Substrate (PIERCE Biotechnology, Rockford, IL, USA) using a LAS-3000 Bioimaging System (Fuji, Duesseldorf, Germany).

### Ethics Statement

The patients reported in this study have provided written consent for the publication of the clinical details and for utilization of their plasma exchange material for experimental studies. The study was approved by the ethics commission of the Medical Faculty of the University of Würzburg (02/06; January, 19^th^ 2006).

Animal use and care were in accordance with the institutional guidelines. All animal experiments were approved by the Bavarian State authorities (Regierung von Unterfranken, # 55.5-2531.01-78/05 and # 55.5-2531.01-12/10).

### Intrathecal passive transfer of IgG fractions

Twenty-two female Lewis rats were used (6–8 weeks old, purchased from Harlan-Winkelmann, Borchen, Germany). Intrathecal catheters (0.28-mm inner diameter; 0.61-mm outer diameter; intrathecal length: 7.0 cm), were placed in the subarachnoid space following the method of Yaksh and Rudy [35]. After allowing rats to recover for 5 days, animals were randomized to receive either purified patient IgG containing antibodies against GAD65 (SPS IgG, concentration 100 mg/ml, n = 6), the purified IgG fraction from a control patient (control IgG, 100 mg/ml, n = 9), and normal saline (n = 7). I.th. injections of 10 µl of patient IgG (containing 1 mg of IgG) or normal saline with a subsequent flush of 10 µl saline were performed daily for 5 days, and q.o.d. for the next 5 injections, and then every three days for the final two injections. Post mortem examinations were done on all rats to exclude catheter-induced spinal cord inflammation.

### Behavioral analysis

Behavioral analyses were done by the same group of investigators with longstanding experience in rat behavioral studies; these were kept blinded as to treatment allocation. Animals were observed daily in their cages and on a plane surface with tunnels and obstacles. Rats were trained on an accelerating RotaRod (TSE Systems, Bad Homburg, Germany) and quantitative testing was performed after recovery from surgery and before the 1^st^ injection (baseline) and on day 4 and 19 after starting IgG injections. Forelimb grip strength was tested with a digital grip force meter (Chatillon, Greensboro, NC, USA). Gait analysis [36] was done on day 5 and 20 after the first injection. Activity levels were tested in an open field box (82×82×25 cm). The arena was evenly illuminated with 100 lux at floor level. The area of the open field was divided into a 65×65 cm central zone and a surrounding border zone. Rats were individually placed into a corner of the open field, and behavior was measured over a 5 min period with a digital video-tracking system (VideoMot2; TSE, Bad Homburg, Germany). Horizontal locomotor activity was determined by the total distance traveled. Anxiety related behavior was analyzed with an elevated plus maze-test. The device comprised two opposing open arms (50×10 cm) and two opposing closed arms (60×10 cm) that had 30 cm high, non-transparent walls. The four arms were connected by a central platform (10×10 cm). The maze was elevated 72 cm above the floor. The open arms were illuminated with an intensity of 100 lux, the central area with 70 lux and the closed arms with 50 lux. Rats were initially placed in the center of the maze facing one of the open arms and then were allowed to investigate the area for 5 min. Their behavior was recorded by video-tracking (VideoMot2). Entry into an arm was defined when the rat moved entirely into the arm. The time spent in the open and closed arms, the number of entries made into them and the time staying in the center were measured to assess anxiety-related behavior in SPS-IgG treated vs. control rats. The total distance traveled during the test session was recorded as a locomotion index [37]. To avoid learning and habituation effects, open field and elevated plus maze testing was only performed at the end of the experiment after completing the i.th. injections (day 19 after starting IgG injections).

### *In-vivo* electrophysiological analysis of spinal cord disinhibition

H-reflex testing: A total of 22 rats were used for these experiments. Under anesthesia with i.p. injections of ketamin and xylazin (80–100 mg/kg and 5 mg/kg, respectively), H-Reflex recording was performed as described previously [38] using a Toennies electromyography unit amplifier and NeuroScreen^plus^ software (Erich Jaeger GmbH, Hoechberg, Germany). For analysis of post-activation depression, the ratio of H-wave over the motor potential amplitudes (H/M-ratio) was determined by repeated single supramaximal stimuli of the tibial nerve at frequencies ranging from 0.1 Hz to 10 Hz [38]. H/M values of the 10^th^ stimulation were taken for further analysis. For analysis of the inhibition after heteronymous stimulation (D1/D2 inhibition), the peroneal nerve was stimulated supramaximally (conditioning volley) before the tibial stimulation at defined latencies (0 ms, 25 ms, 50 ms, 75 ms, 100 ms, 150 ms, 200 ms, and 500 ms), determined by a Master-8 stimulator (A.M.P.I., Jerusalem, Israel).

Dorsal root potential (DRP) recordings: lumbar laminectomy was performed to expose spinal segments TH12-L5, and DRP were recorded from dorsal roots L4 and 5 on both sides with an ELC-03X amplifier (npi electronic, Tamm, Germany) after stimulation of the tibial nerve using a Grass S88 stimulator (duration 0.2 ms, single stimulation and train of 3 stimuli at 100 Hz; Grass technologies, RI, USA) as described previously [39], [40]. In a subset of animals the dependence of the DRP amplitude on GABAergic transmission was tested by locally applying bicucullin to the dorsal root entry zone of the spinal cord.

### Immunohistochemical analysis and confocal imaging

At the end of the experiments, rats were deeply anesthetized with intraperitoneal injections of ketamin and xylazin (80–100 mg/kg and 5 mg/kg, respectively) and blood was withdrawn. Thereafter, the rats were sacrificed, and the lumbar spinal cord and the brain were mounted in Tissue-Tec OTC embedding compound, and deep frozen. Twenty-µm cryosections of the brain hemispheres were cut and serial hematoxylin and eosin staining was performed to identify the brain regions with the structures of interest according to a rat brain atlas [41]. Sections were incubated at 4°C over night with polyclonal rabbit anti-human IgG (Dako, Hamburg, Germany; 1∶200) using Cy3 (Dianova, Hamburg, Germany, 1∶100) as fluorochrome (2 hours at room temperature). Brain sections were viewed with a Zeiss Pascal 5 microscope using a 63x oil immersion lens (Zeiss, Oberkochen, Germany) with the relevant filter settings.

To test the binding properties of the patient plasma filtrate and purified IgG, we used 20-µm frozen sections of brain and lumbar spinal cord of naive rats and, in addition, 20-µm frozen sections from frontal cortex, amygdala, and spinal cord of human control autopsy material without CNS disease. Sections were incubated with patient plasma exchange material in concentrations ranging from 1∶100 to 1∶10.000 and with purified patient IgG in concentrations of 100 µg/ml and 10 µg/ml over night as the primary antibody, followed by rabbit anti-human IgG (Dako) at 1∶200 for 30 minutes and visualized with diaminobenzidine.

Besides binding we were interested in potential cell destruction, complement activation and immune cell infiltration. We incubated 20-µm frozen brain sections at 4°C over night with a rabbit monoclonal anti-capase-3 antibody (BD Biosciences, San Jose, USA, 1∶200), a rabbit polyclonal anti-C5b-9 antibody (abcam, Cambridge, UK, 1∶1000), a mouse monoclonal anti-rat CD68 antibody (Linaris, Wertheim-Bettingen, Germany, 1∶000) and a mouse monoclonal anti-CD3 antibody (BD Biosciences, San Jose, USA, 1∶200). Immunoreactions were then visualized with diaminobenzidine.

### High performance liquid chromatography (HPLC) analysis of GABA release

Hippocampal cell cultures were prepared from E18 mouse embryos as described previously [42], cultured for 10 days and were then incubated with SPS patient IgG and control IgG. After 6 hours, medium was removed and neurons were preincubated for 90 s at 37°C with 200 µl of 119 mM NaCl, 2.5 mM KCl, 2 mM CaCl_2_, 2 mM MgCl_2_, 25 mM Hepes and 30 mM glucose (ACSF). Thereafter, cell cultures were either stimulated by depolarization with 200 µl of a high potassium containing solution (31.5 mM NaCl, 90 mM KCl, 2 mM CaCl_2_, 2 mM MgCl_2_, 25 mM Hepes and 30 mM glucose) or 200 µl ACSF for 90s at 37°C (n = 6 for each condition) [43], [44]. GABA concentrations were analyzed in the supernatants by HPLC with fluorescence detection, employing the precolumn derivation method with ortho-phthaldialdehyde with an automated HPLC system (Agilent 1100 Series, Agilent, Waldbronn, Germany) as described previously [45]. The excitation and emission wavelengths of the fluorescence detector were set at 330 and 450 nm, respectively. The HPLC investigator was blinded as to treatment allocation.

### Statistical analysis

Statistical analyses were done using SPSS software (SSPS Inc.). The data represent means +/− SEM. The significance of differences in means was tested using students't-test or Mann-Whitney-U-test depending on the distribution of data.

## Supporting Information

Figure S1 **Intrathecal passive transfer of purified SPS IgG does not affect spinal GABAergic presynaptic inhibition.** (A and B) *In-vivo* recording of the H-reflex in rats intrathecally treated with purified SPS patient IgG (n = 6), control IgG (n = 9) or saline (n = 7). Spinal inhibition was assessed by measuring the decrease of H-reflex amplitude at different stimulus frequencies (post-activation depression (A)) and after preceding heteronymous stimulation of a nerve supplying antagonistic muscles (D1/D2 inhibition (B)). Both test paradigms revealed no significant differences between treatment with SPS IgG and control animals with regular inhibition of the H-reflex amplitude, indicating unaffected spinal polysynaptic inhibition (one-way ANOVA). (C) Direct analysis of the spinal GABAergic presynaptic inhibition was performed by *in-vivo* recording of the dorsal root potential (DRP) peak amplitude. Intrathecal treatment with SPS IgG did not result in a decrease of DRP amplitudes compared with controls and also temporal summation after high frequent repetitive stimulation was not different between the experimental groups, indicating normal GABAergic spinal inhibition upon i.th. passive transfer(one-way ANOVA).
(TIF)
