# Supplementary figures and images for: Human Stiff-Person Syndrome IgG Induces Anxious Behavior in Rats
Source: PLoS One. 2011 Feb 8;6(2):e16775. doi: 10.1371/journal.pone.0016775 (PMC3035624; doi:10.1371/journal.pone.0016775)

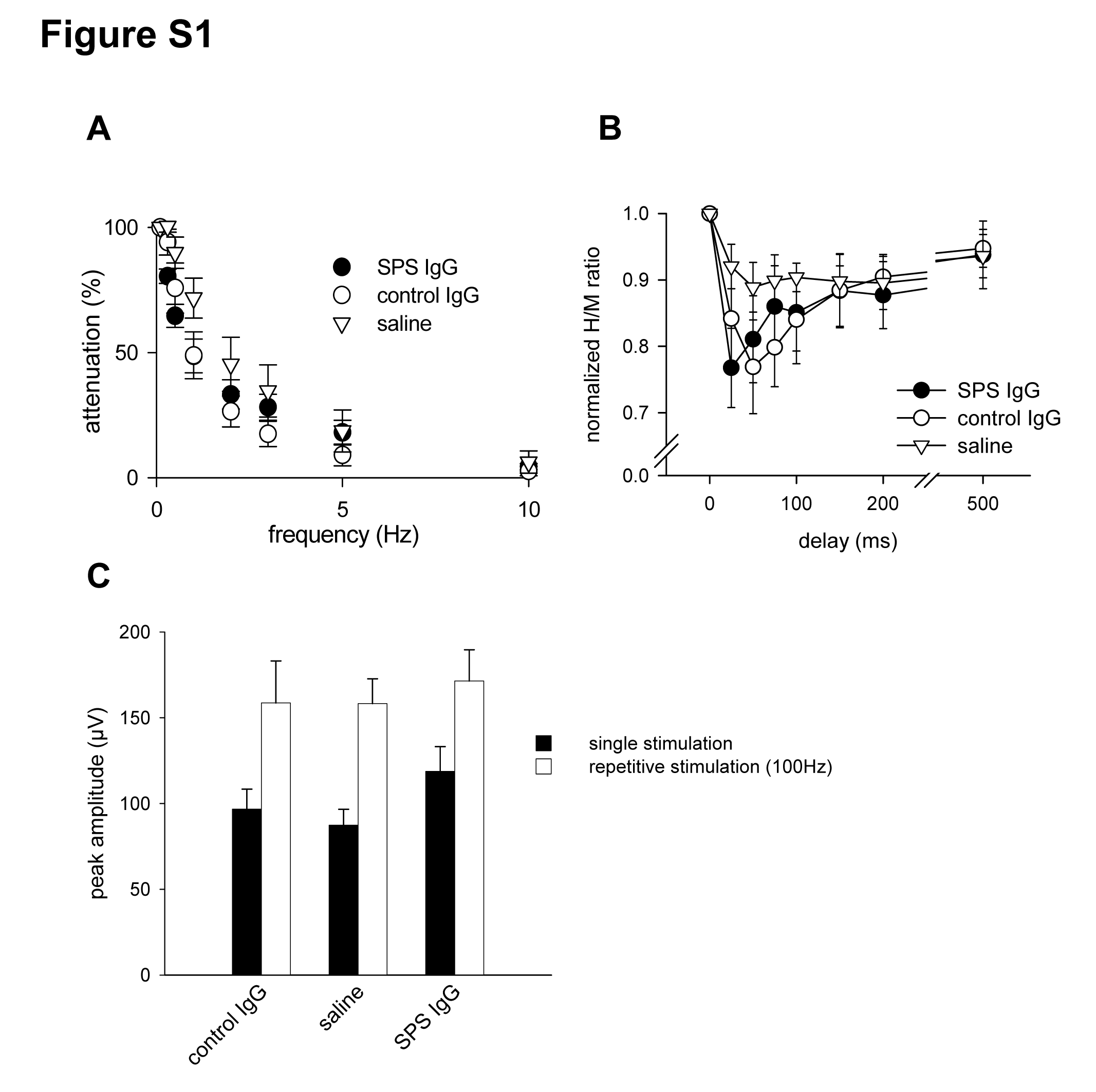

Supplement: Figure S1 — Intrathecal passive transfer of purified SPS IgG does not affect spinal GABAergic presynaptic inhibition. (A and B) In-vivo recording of the H-reflex in rats intrathecally treated with purified SPS patient IgG (n = 6), control IgG (n = 9) or saline (n = 7). Spinal inhibition was assessed by measuring the decrease of H-reflex amplitude at different stimulus frequencies (post-activation depression (A)) and after preceding heteronymous stimulation of a nerve supplying antagonistic muscles (D1/D2 inhibition (B)). Both test paradigms revealed no significant differences between treatment with SPS IgG and control animals with regular inhibition of the H-reflex amplitude, indicating unaffected spinal polysynaptic inhibition (one-way ANOVA). (C) Direct analysis of the spinal GABAergic presynaptic inhibition was performed by in-vivo recording of the dorsal root potential (DRP) peak amplitude. Intrathecal treatment with SPS IgG did not result in a decrease of DRP amplitudes compared with controls and also temporal summation after high frequent repetitive stimulation was not different between the experimental groups, indicating normal GABAergic spinal inhibition upon i.th. passive transfer(one-way ANOVA). (TIF) [file pone.0016775.s001.tif]
